# Arabic translation, adaptation and modification of the dialysis symptom index for chronic kidney disease stages four and five

**DOI:** 10.1186/s12882-015-0036-2

**Published:** 2015-03-27

**Authors:** Hayfa Almutary, Ann Bonner, Clint Douglas

**Affiliations:** School of Nursing, Queensland University of Technology, Victoria Park Rd, Kelvin Grove, Brisbane, QLD 4059 Australia; School of Nursing, King Abdulaziz University, Jeddah, Saudi Arabia; Chronic Kidney Disease Centre for Research Excellence, University of Queensland, Brisbane, Australia; Visiting Research Fellow, Renal Medicine, Royal Brisbane and Women’s Hospital, Brisbane, Australia

**Keywords:** Chronic kidney disease, Symptom burden, Dialysis, Non-dialysis, Instrument

## Abstract

**Background:**

Symptom burden in chronic kidney disease (CKD) is poorly understood. To date, the majority of research focuses on single symptoms and there is a lack of suitable multidimensional symptom measures. The purpose of this study was to modify, translate, cross-culturally adapt and psychometrically analyse the Dialysis Symptom Index (DSI).

**Methods:**

The study methods involved four phases: modification, translation, pilot-testing with a bilingual non-CKD sample and then psychometric testing with the target population. Content validity was assessed using an expert panel. Inter-rater agreement, test-retest reliability and Cronbach’s alpha coefficient were calculated to demonstrate reliability of the modified DSI. Discriminative and convergent validity were assessed to demonstrate construct validity.

**Results:**

Content validity index during translation was 0.98. In the pilot study with 25 bilingual students a moderate to perfect agreement (Kappa statistic = 0.60-1.00) was found between English and Arabic versions of the modified DSI. The main study recruited 433 patients CKD with stages 4 and 5. The modified DSI was able to discriminate between non-dialysis and dialysis groups (*p* < 0.001) and demonstrated convergent validity with domains of the Kidney Disease Quality of Life short form. Excellent test-retest and internal consistency (Cronbach’s α = 0.91) reliability were also demonstrated.

**Conclusion:**

The Arabic version of the modified DSI demonstrated good psychometric properties, measures the multidimensional nature of symptoms and can be used to assess symptom burden at different stages of CKD. The modified instrument, renamed the CKD Symptom Burden Index (CKD-SBI), should encourage greater clinical and research attention to symptom burden in CKD.

**Electronic supplementary material:**

The online version of this article (doi:10.1186/s12882-015-0036-2) contains supplementary material, which is available to authorized users.

## Background

Chronic kidney disease (CKD) is associated with a wide range of physical and psychological symptoms which have a negative impact on health-related quality of life [1]. Fatigue, pain, pruritus, sleep disturbances and decreased appetite are some of the most prevalent and frequently reported symptoms in CKD patients [2] and are the main predictors of increased morbidity and mortality [3,4]. Alleviating or controlling symptoms in CKD patients are one of the main priorities of the renal multidisciplinary team and thus require comprehensive assessment [5]. A major impediment to understanding the symptom experience in CKD is the lack of suitable measures that examine a wide range of symptoms across key dimensions (i.e. prevalence, severity, frequency and distress). Symptom prevalence alone does not reflect the complete patient symptom experience. The symptom experience is much better understood in cancer patients as a multidimensional burden comprising prevalence, distress, severity and frequency [6,7]. Though CKD patients often have greater prevalence and distress than advanced cancer patients [2], severity and frequency are rarely measured.

The Dialysis Symptom Index (DSI) is the most frequently used measure to assess symptoms in CKD and end stage kidney disease [2]. Developed by Weisbord et al. [8], the DSI was designed to examine the prevalence and distress of 30 self-reported physical and emotional symptoms. This contrasts with the purposes of other available instruments, such as the Modified Edmonton Symptom Assessment System [9], the Patient Outcome Scale Symptom Module (POS-S) [10] and the Pittsburgh Symptom Score [11] that have been used to assess symptom burden in CKD patients. However, none of the currently available instruments measure the multidimensional nature of symptoms [12,13].

The ideal CKD symptom assessment measure should assess a range of symptoms and be able to determine the prevalence, distress, severity and frequency of symptoms [2]. The purpose of this study was to modify, translate, culturally adapt, and then evaluate the psychometric properties of the Arabic version of the DSI. It was part of a larger study examining symptom clusters in people with CKD.

## Methods

The psychometric property testing involved four phases: modification of the DSI, translation, pilot study and the main study.

### Phase 1 - Instrument modification

Permission was granted by Weisbord to modify and then translate the DSI into Arabic. First, two symptoms (depression and nocturia) and three free fields were added. Both depression [14] and nocturia [15,16] are highly prevalent in CKD, but are not routinely included in symptom measures. The additional free fields were included to capture any possible symptoms that were missing from the DSI. Then two symptom dimensions (severity and frequency) were also added to the DSI [2,12,13]. All symptom dimensions (frequency, severity and distress) were rated on a 0-10 point scale because this type of response scale is easier for patients to use (i.e. similar to the commonly used numerical pain rating scale), provides a wider range of possible scores, and increases the statistical analyses that are available. In the original DSI, patients were asked to consider symptoms they had experienced in the previous week. However, we extended the time frame to four weeks to reflect the chronic nature of CKD symptoms. For example, some symptoms may occur often only during dialysis (e.g. cramps and dizziness) and may not be persistent over 4 weeks. These modifications created a comprehensive instrument, re-named (with permission from Weisbord) the CKD Symptom Burden Index (CKD-SBI), to assess symptoms in different stages of CKD. The time needed to complete the modified instrument ranged between 15 to 30 minutes.

The CKD-SBI includes four symptom dimensions (prevalence, distress, severity and frequency) across 32 symptoms (see Additional file 1). The prevalence scale assesses the presence or absence (Yes/No) of each symptom with a higher overall score indicating a higher symptom occurrence. The possible range of prevalence scores is 0-32. Distress, severity and frequency are each measured using a 0 to 10 scale for each of the symptoms. Participants rate the “distress” subscale from *none* to *very much*, “severity” from *none* to *very severe*, and “frequency” from *never* to *constant*. A maximum total score for each of these scales is 320 (range 0-320). The total CKD-SBI score is calculated by summing the scores of each CKD-SBI subscale (prevalence, distress, severity and frequency) and then multiplying the result by 0.1008 (constant number). The total score for the CKD-SBI scale therefore ranges between 0-100, where 100 indicates the highest symptom burden possible.

### Phase 2 - Translation procedure

**Figure 1:**
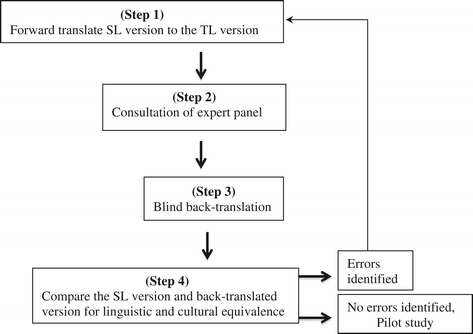
**Steps of Brislin’s method of translation application.**

We used the Brislin back-translation method [14,17]; the most frequently used and accepted method for effective translation equivalence of instruments [18]. The method begins with a bilingual expert translating the instrument from its source language (English) into the target language (Arabic). Consultations with an expert panel are an important stage to ensure clarity, detect linguistic mistakes and ensure cross-cultural equivalence [19]. Then a blind back-translation (without accessing the original source language version) from the target language to the source language should be undertaken by another bilingual translator [18]. The back-translated version is then compared with the original version. If there are errors in the meaning in the blind back-translated version after comparison with the original version, the process of translation is repeated for the sections that include errors in the meaning [20]. Figure 1 summarises the steps in the Brislin’s method.

Translation processes were undertaken in Saudi Arabia using bilingual (Arabic/ English), certified translators. Formal written Arabic language was used as this form of Arabic is used in all Arabic-speaking countries; thus increasing the usefulness of the instrument to other CKD populations outside of Saudi Arabia. The Arabic expert panel involved three nephrology nurses, two nephrologists, a social worker and two primary school teachers. The goal of the panel was to: 1) ensure clarity, readability and linguistic appropriateness; 2) review of grammatical style and comprehensibility; and 3) maintain cultural equivalence. The primary school teachers ensured suitable literacy level of the CKD-SBI for people with limited literacy skills (which will be tested in the pilot study). The expert panel recommended several minor linguistic and grammatical modifications, correction of spelling, and clearer instruction statements. A blind back-translation from the Arabic version into English was then performed by another bilingual certified translator independently of the first translator and without access to the original English CKD-SBI. The procedure that was used to compare the back-translated version with the original version involved a new independent professional translator, two bilingual members of the expert panel and the first author. There were no errors identified in meaning after comparing both versions.

Content validity index (CVI) testing also occurred during this phase using the expert panel and a professional translator from a Saudi hospital. Individually they were asked to rate their level of agreement with the relevance and equivalence of each item between the Arabic and English versions on a four-point scale (1 = ‘not relevant’ to 4 = ‘very relevant’) to evaluate content validity [21]. A score of 3 or 4 indicates that the translated item is valid and equivalent to the English version [21]. The total CVI was 0.98 with items ranging from 0.88-1.00, which indicated that the CKD-SBI had good content validity.

### Phase 3 - Pilot study

Siaki [22] recommends pretesting of a translated instrument as a method to determine equivalence between the target language and source language. We conducted a pilot study of both versions of the CKD-SBI using bilingual university students and measured test-retest reliability, intra-rater reliability and internal consistency. As symptoms are also experienced by normal healthy populations but in a lesser frequency than those with CKD, using university students was appropriate when assessing language equivalence. Several studies have used this method when assessing language equivalence [23-25]. The pilot study involved anonymous completion of the English and Arabic versions of the CKD-SBI, on two separate occasions, one week apart. Demographic information (i.e. age, gender) was not relevant to determining equivalence and was not collected. An Arabic announcement was applied to the university notice board, and a snowball sampling technique was used to recruit participants during April-May, 2013. Ethics approval was obtained from the Queensland University of Technology (QUT) prior to commencement.

### Phase 4 - Main study

The main study used a cross-sectional design to evaluate the psychometric properties of the CKD-SBI in Arabic-speaking CKD patients who were either receiving dialysis or not. A convenience sample of CKD patients from three hospitals in Saudi Arabia were recruited during July 2013 to February 2014. Initially treating clinicians identified potential eligible participants - adults with CKD (eGFR < 30 mls/min/1.73 m^2^) or currently receiving either haemodialysis or peritoneal dialysis, willing to participate, and able to communicate in Arabic. Exclusion criteria were cognitive impairment that would preclude voluntary, informed consent, and those with critical conditions. Ethical approval was obtained from King Abdulaziz University Hospital, Jeddah Research Centre, and QUT Human Research Ethics Committees. All participants provided written informed consent. Data was collected through in-person distribution in the nephrology clinics (non-dialysis CKD patients) and the kidney dialysis centres (dialysis patients). Participants completed the newly translated Arabic version of the CKD-SBI (32 items) along with the Kidney Disease Quality of Life short form (KDQOL-36™). The KDQOL-36™ has 36 items and is available in Arabic [26]. The total score of the KDQOL-36 ranges from 0-100, with higher scores indicating a better QOL. Demographic and renal characteristics were also collected. Davies et al.’s [27] co-morbidity index was used to estimate the number of co-morbidities for the sample. This index consists of seven domains of co-morbid conditions, including malignancy (active, non-cutaneous); ischaemic heart disease, peripheral vascular disease, left ventricular dysfunction, diabetes mellitus (type 1 or 2); systemic collagen vascular disease; or other significant pathology. The total score is determined by summing the score of each co-morbid domain. The scores are then graded into three risk groups: grade 0 is a zero score, grade 1 (1-2 domains), or grade 2 (3-7 domains).

### Statistical analysis

Data for the pilot study and the main study were entered into IBM SPSS Statistics version 21 in two different data sets and reported in the results section separately. For the pilot study, the percentage of agreement between the original English and Arabic versions of the CKD-SBI was calculated to examine equivalence. Kappa statistics were used to assess the inter-rater agreement between the English and Arabic versions for categorical data. Intra Class Correlation coefficient and Wilcoxon signed-ranks test for matched pairs (numerical scales) were used to determine the consistency of CKD-SBI scores. The ICC coefficient was used to estimate the inter-rater reliability of the CKD-SBI. Pearson’s correlation coefficients were also calculated to assess the test-retest reliability. Cronbach’s alpha reliability coefficients were calculated to determine the internal consistency.

For the main study, descriptive statistics were used to summarise sample characteristics. Cronbach’s alpha reliability coefficients were calculated to determine the internal consistency of the CKD-SBI. Pearson’s correlations were also used to examine associations between symptom dimensions. Discriminative validity was assessed using Welch t-tests to assess if the CKD-SBI was able to discern between known groups according to disease severity within the CKD population. For convergent validity, Pearson correlation coefficients were used to examine the relationships between the symptom subscales of the CKD-SBI and all domains of KDQOL-36™. Significance was set at *p* < 0.05.

## Results

### Pilot study

The pilot study involved 25 bilingual students, producing 25 matched English (E)/Arabic (A) pairs. There was a high percentage of agreement between E and A versions across all the items with a mean of 98.45 ± 3.04 (range = 88-100%). Level of agreement between the E and A versions revealed Kappa (κ) scores ranging from 0.603 – 1.0. The strength of agreement is considered poor when κ < 0.20, fair between 0.21 and 0.40, moderate between 0.41 and 0.60, substantial between 0.61 and 0.80, and excellent when κ > 0.80 [28]. Most items (23 out of 31) revealed a κ of 1.00 indicating complete agreement (see Table 1). The ICC score indicated an excellent agreement between the E and A versions of the CKD-SBI (ICC = 0.99, *p* < 0.001). In addition, the Wilcoxon signed rank test was used to examine the difference between the E and A versions for the total CKD-SBI scale and for each subscale (prevalence, distress, severity and frequency). The results revealed no significant differences between the E and A versions of the CKD-SBI for the total scale of CKD-SBI (Z = 1.79, *p* = 0.07), as well as for each subscale: prevalence (Z = 1.34, *p* = 0.18), distress (Z = 1.14, *p* = 0.26), severity (Z = 1.83, *p* = 0.68) and frequency (Z = 1.48, *p* = 0.14).

**Table 1:** **Percentage of equivalence and Kappa statistics for English and Arabic CKD Symptom Burden Index for the pilot study (** ***N*** **= 25)**

**Symptom**	**% of agreement**	**K (** ***p*** **)**
1. Constipation	92	0.841 (<0.001)
2. Nausea	100	1.00 (<0.001)
3. Vomiting	96	0.896 (<0.001)
4. Diarrhoea	100	1.00 (<0.001)
5. Decreased appetite	100	1.00 (<0.001)
6. Muscle cramps	100	1.00 (<0.001)
7. Swelling in legs	100	1.00 (<0.001)
8. Shortness of breath	100	1.00 (<0.001)
9. Light headedness or dizziness	100	1.00 (<0.001)
10. Restless legs or difficulty keeping your legs still	96	0.834 (<0.001)
11. Numbness or tingling in feet	100	1.00 (<0.001)
12. Feeling tired or lack of energy	100	1.00 (<0.001)
13. Cough	100	1.00 (<0.001)
14. Dry mouth	100	1.00 (<0.001)
15. Bone or joint pain	100	1.00 (<0.001)
16. Chest pain	100	1.00 (<0.001)
17. Headache	96	0.779 (<0.001)
18. Muscle soreness	100	1.00 (<0.001)
19. Difficulty concentrating	100	1.00 (<0.001)
20. Dry skin	100	1.00 (<0.001)
21. Itching	100	1.00 (<0.001)
22. Worrying	96	0.803 (<0.001)
23. Feeling nervous	96	0.826 (<0.001)
24. Trouble falling asleep	92	0.828 (<0.001)
25. Trouble staying asleep	100	1.00 (<0.001)
26. Feeling irritable	100	1.00 (<0.001)
27. Feeling sad	100	1.00 (<0.001)
28. Feeling anxious	88	0.603 (0.001)
29. Depression	100	1.00 (<0.001)
30. Decreased interest in sex	100	1.00 (<0.001)
31. Difficulty becoming sexually aroused	100	1.00 (<0.001)
32. Nocturia	100	1.00 (<0.001)

Pearson’s correlation coefficients assessed test-retest reliability and revealed a strong positive correlation between the mean scores for both versions of the CKD-SBI (*r* = 0.997, *p* < 0.01). A Cronbach’s alpha coefficient of 0.7 is generally accepted to estimate the internal consistency of a new scale [29]. Cronbach’s alpha coefficients for the E version (Cronbach’s α = 0.897) and the A version (Cronbach’s α = 0.899) both demonstrated good internal consistency.

### Main study

#### Sample characteristics

A total of 433 patients with CKD were recruited. The mean age was 48.23 ± 14.89 years, 52.9% were male, and 58.4% had at least one co-morbid condition. Most of the participants were receiving dialysis treatment (*n* = 329, 76%). Diabetes and hypertension were the leading causes of CKD in our sample. The overall mean of KDQOL-36 score was 58.08 ± 20.04, ranging from 6.88 to 99. Sample characteristics are summarised in Table 2.

**Table 2:** **Sample characteristics for the main study (** ***N*** **= 433)**

**Characteristics**	***n***	***%***
Age (mean, SD)	48.23	14.89
Gender		
Male	229	52.9
Female	204	47.1
Marital status		
Married	257	59.4
Not married	175	40.4
Treatment type		
Dialysis	329	76
Non-dialysis (eGFR 5-29 mL/min/1.73 m^2^)	104	24
Causes of CKD		
Diabetic nephropathy	135	31.2
Hypertensive nephropathy	146	33.7
Primary glomerular disease	30	6.9
Unknown aetiology	59	13.6
Others	63	14.5
Comorbid conditions		
0	180	41.6
1-2	223	51.5
≤3	30	6.9

Percentages vary depending on missing data.

#### CKD-SBI psychometric results

The total mean CKD-SBI score was 17.98 ± 16.11, ranging from 0 to 83.36. Symptoms were highly prevalent in this sample of CKD patients (mean = 12.58 ± 7.9; range 0-32). As the purpose of this article is to present the psychometric analysis of the CKD-SBI further descriptive results are not required and will be presented in another separate paper. Summary statistics for each subscale of CKD-SBI are presented in Table 3. There was a high internal consistency of the overall CKD-SBI (Cronbach’s α = 0.91) and subscales (range = 0.93 - 0.90). There were strong positive intercorrelations between CKD-SBI subscales, which ranged from 0.82 - 0.97 (see Table 3).

**Table 3:** **Descriptive statistics, reliability and Pearson’s correlation coefficient of the CKD-SBI subscales**

**Subscale**	**Mean**	**SD**	**Range**	**α**	**1**	**2**	**3**	**4**	**5**
1. Prevalence	12.58	7.89	0-32	0.924	-				
2. Distress	55.47	51.74	0-259	0.904	0.817	-			
3. Severity	57.56	52.26	0-265	0.924	0.835	0.963	-		
4. Frequency	58.76	50.48	0-273	0.929	0.838	0.943	0.967	-	
5. Total symptom burden	17.84	15.98	0-83	0.91	0.857	0.981	0.991	0.983	-

#### Discriminative validity

Discriminative validity is the ability of an instrument to distinguish between groups that are expected to differ based on their clinical diagnosis or other characteristics [30]. It is an important measure of construct validity, which is commonly examined through the “known groups” technique [30]. As renal function deteriorates, symptoms become more prevalent due to the accumulation of uraemic toxins [31]. Thus we divided the sample into two groups (dialysis and non-dialysis patients) and compared the total symptom burden score. We hypothesised that symptom burden would increase as GFR decreases. Due to the unequal sample size, Welch t-tests were used to compare groups [32]. This showed significant differences in total symptom burden scores (*t* (403.5) = -13.1, *p* < 0.001), indicating that the CKD-SBI is able to discriminate between groups that have different eGFR levels.

#### Convergent validity

Convergent validity refers to the degree to which scores on a measure associate with scores on other measures that are intended to assess similar constructs [33]. Previous studies have shown that symptoms among CKD patients are correlated with poor health-related quality of life [34,35]. Therefore, we hypothesised that all symptom dimension scores would be negatively correlated with domain scores of the KDQOL-36. Pearson’s correlation coefficients demonstrated moderate to strong relationships in the expected direction between all symptom dimensions (prevalence, distress, severity and frequency) and each domain of the KDQOL-36 (see Table 4).

**Table 4:** **Pearson correlations between CKD-SBI symptom dimension subscales and KDQOL-36 domains (** ***N*** **= 433)**

**CKD-SBI**	**KDQOL-36**					
	**Total KDQOL scale score**	**Physical function summary**	**Mental health summary**	**Effects of kidney disease on daily life**	Symptoms and problems subscale*	**Burden of the disease**
Prevalence	*r* = -0.58**	*r* = -0.51**	*r* = -0.41**	*r =* -0.47**	*r =* 0.72**	*r =* -0.33**
Distress	*r* = -0.63**	*r* = -0.51**	*r* = -0.44**	*r* = -0.54**	*r =* 0.78**	*r* = -0.36**
Severity	*r* = -0.61**	*r* = -0.49**	*r* = -0.43**	*r* = -0.51**	*r =* 0.77**	*r* = -0.34**
Frequency	*r* = -0.61**	*r* = -0.51**	*r* = -0.44**	*r* = -0.49**	*r =* 0.75**	*r* = -0.35**
Total symptom burden	*r* = -0.63**	*r* = -0.52**	*r* = -0.44**	*r* = -0.53**	*r* = 0.75**	*r* = -0.44**

*The results are presented after reverse scoring the symptoms and problems subscale of the KDQOL-36 to be consistent and in the same direction with our scoring system.

**Correlation is significant at the 0.01 level (2-tailed).

## Discussion

This study modified, translated, culturally adapted and psychometrically evaluated the CKD-SBI. Jablonski [12] argues that when examining CKD symptom burden, the multidimensional nature of symptoms should be considered. However, a significant criticism of research in this area is the lack of comprehensive CKD symptom measures. The DSI was selected as a target instrument in this study for two reasons. First, there is no disease-specific instrument in Arabic for assessing CKD symptoms. Second, we believed that the DSI is the best available instrument for symptom assessment but it required some modifications to be more comprehensive to assess the multidimensional aspects of symptoms and gain better understanding of symptom burden.

The results demonstrated that the CKD-SBI was linguistically and culturally relevant to CKD patients from Arabic-speaking backgrounds. In the translation process, no statements required adjustment to fit into the Arabic cultural context. Excellent content validity of the Arabic version of the CKD-SBI was demonstrated. Since formal written Arabic language is universally understood amongst Arabic speaking nations, this instrument is applicable for other populations. The DSI was originally developed and validated for patients receiving dialysis treatment. After modification, testing and with approval from the DSI originator, we renamed the DSI as the CKD Symptom Burden Index (CKD-SBI), to reflect the utility of the instrument to measure symptom burden and to broaden its appeal to other researchers to assess symptoms in the non-dialysis CKD populations.

In this study, symptoms were highly prevalent among CKD patients with an average of 12.58 symptoms per patient. This finding is consistent with a recent comprehensive review of 19 studies, which found the mean symptom per patients ranged between 6-20 symptoms [2]. Based on literature, symptoms in CKD were often described as a part of KDQOL [2]. However, the KDQOL does not measure the most prevalent symptoms reported in the literature; that is fatigue, bone or joint pain, and psychological symptoms. This suggests that using the symptom list of the KDQOL is not sufficient measure symptom burden in CKD patients. The results from this study emphasise using a comprehensive symptom measure to examine symptom burden among CKD patients.

Overall the results provide initial evidence of validity and reliability of the CKD-SBI, which has potential clinical and research implications. One of the strengths of the main study was having a large sample size and recruiting CKD patients from three different hospitals. Sample characteristics in this study were similar to population data reported by the Saudi Center for Organ Transplantation [36] and a recent review on CKD in Saudi Arabia [37], which provides some evidence that participants were representative of the target population. We also administered the scale to patients with different treatment modalities and stages of CKD. Good discriminant validity was demonstrated, indicating that the CKD-SBI can discriminate between patients in stages 4 and 5. A consistent technique (in-person distribution by one person [first author]) was used to administer the instrument. This technique assisted with providing insight into the clinical utility of the instrument. Patients reported acceptability and clarity of the instrument regardless of age and educational level. The time taken to complete it was short (on average 20 minutes) although this was dependent on the number of symptoms experienced.

Further evidence of construct validity was demonstrated by examining relationships between the CKD-SBI and domains of KDQOL-36. These results are consistent with previous studies that have demonstrated CKD symptoms are negatively correlated with KDQOL [9,13,35]. Similarly, Abdel-kader et al. [34] found moderate correlation between the DSI and Physical Component Summary and Mental Component Summary of the quality of life domains using the Medical Outcomes Study Short Form-36. The results suggest that the two instruments, CKD-SBI and KDQOL-36, together demonstrated convergent validity. Used together, they provide clinicians with a comprehensive understanding of CKD symptom burden and relationship with quality of life. The additional information gained from this new multidimensional instrument is better to predict the important patient outcomes such as HRQoL, depression, morbidity, mortality, which is worth the additional respondent burden. However, further work is needed to demonstrate the strong relationships between symptom burden and other important measures of HRQOL, depression, morbidity, and mortality.

### Limitations

The results are significant as this is the first study to validate a comprehensive instrument designed to assess CKD symptom burden. However, there are several limitations that should be acknowledged. It is difficult to identify bilingual patients thus test-retest agreement may have been overestimated by use of the instrument in a generally healthy population (university students). Also, the test-retest reliability was one week apart, thus there was potential for stability bias as participants could have memorised their responses between the two tests [30]. Since this instrument has been validated only in Arabic-speaking people with CKD stage 4 and 5, more research is needed to validate this instrument in English-speaking populations as well as in other CKD stages. In addition, it would have been ideal to compare the CKD-SBI with another multidimensional symptom measure, but another suitable instrument is not yet available. This limits the establishment of criterion-related validity for the CKD-SBI.

## Conclusion

The CKD-SBI demonstrated good psychometric properties and is suitable to assess symptom burden for different CKD populations. It is comprehensive and can capture the multidimensional aspects of a range of symptoms. Assessing symptom burden could help health professionals to have a better understanding of the symptom experience of CKD patients and to identify symptoms that have a major impact on health-related quality of life. The lack of knowledge of the symptom experience and its impact on people’s lives leads to under-assessment and treatment of symptoms. This new multidimensional instrument should encourage greater clinical and research attention to symptom burden in CKD.

## Additional file

Additional file 1:
**Arabic version of CKD-SBI.**
