# Supplementary material for: Arabic translation, adaptation and modification of the dialysis symptom index for chronic kidney disease stages four and five
Source: BMC Nephrol. 2015 Mar 27;16:36. doi: 10.1186/s12882-015-0036-2 (PMC4377038; doi:10.1186/s12882-015-0036-2)
Supplement: Additional file 1: — Arabic version of CKD-SBI. [file 12882_2015_36_MOESM1_ESM.pdf]

Additional file 1: Arabic version of CKD-SBI

مؤشر عبء الأعراض لمرض الفشل الكلوي المزمن

التعليمات

فيما يلي قائمة بالأعراض التي قد توجد لدى الاشخاص الذين يعانون من مرض الفشل الكلوي المزمن. أقراء كل واحدة منها بعناية ، إذا كان لديك أي وحده من هذه الأعراض خلال الأسابيع الأربعة الماضية ، يرجى إخبارنا الى أي **درجة أزعجتك أو ضايقتك** ، مدى **شدتها** في العادة و **عدد المرات** التي تشعر بها و ذلك عن طريق وضع دائرة حول الرقم الملائم.

**خلال الأسابيع الأربعة الماضية:** **هل عانيت من هذا العرض؟**		**إذا نعم:**		
		**الى أي درجة أزعجتك أو ضايقتك على المقياس صفر - ١٠**<br>**صفر = لا يوجد**<br>**١٠ = كثيراً جداً**	**كيف كانت شدتها في العادة على المقياس صفر-١٠؟**<br>**صفر= لا يوجد**<br>**١٠ = شديدة جداً**	**كم مرة يتكرر ذلك على المقياس صفر-١٠؟**<br>**صفر= أبداً**<br>**١٠ = تقريبا بشكل مستمر**
**١- الإمساك**	**لا**<br>نعم ←	٠ ١ ٣ ٢ ٤ ٦ ٥ ٧ ٨ ٩ ١٠	٠ ١ ٣ ٢ ٤ ٦ ٥ ٧ ٨ ٩ ١٠	٠ ١ ٣ ٢ ٤ ٦ ٥ ٧ ٨ ٩ ١٠
**٢- الغثيان**	**لا**<br>نعم ←	٠ ١ ٣ ٢ ٤ ٦ ٥ ٧ ٨ ٩ ١٠	٠ ١ ٣ ٢ ٤ ٦ ٥ ٧ ٨ ٩ ١٠	٠ ١ ٣ ٢ ٤ ٦ ٥ ٧ ٨ ٩ ١٠
**٣- الاستفراغ**	**لا**<br>نعم ←	٠ ١ ٣ ٢ ٤ ٦ ٥ ٧ ٨ ٩ ١٠	٠ ١ ٣ ٢ ٤ ٦ ٥ ٧ ٨ ٩ ١٠	٠ ١ ٣ ٢ ٤ ٦ ٥ ٧ ٨ ٩ ١٠
٤- الاسهال	**لا**<br>نعم ←	٠ ١ ٣ ٢ ٤ ٦ ٥ ٧ ٨ ٩ ١٠	٠ ١ ٣ ٢ ٤ ٦ ٥ ٧ ٨ ٩ ١٠	٠ ١ ٣ ٢ ٤ ٦ ٥ ٧ ٨ ٩ ١٠
٥- فقدان الشهية	**لا**<br>نعم ←	٠ ١ ٣ ٢ ٤ ٦ ٥ ٧ ٨ ٩ ١٠	٠ ١ ٣ ٢ ٤ ٦ ٥ ٧ ٨ ٩ ١٠	٠ ١ ٣ ٢ ٤ ٦ ٥ ٧ ٨ ٩ ١٠

**خلال الأسابيع الأربعة الماضية:** **هل عانيت من هذا العرض؟**		**إذا نعم:** الى أي درجة أزعجتك أو ضايقتك على المقياس صفر ـ ١٠ صفر = لا يوجد ١٠ = كثيراً جداً	**إذا نعم:** كيف كانت شدتها في العادة على المقياس صفر-١٠؟ صفر= لا يوجد ١٠ = شديدة جداً	**إذا نعم:** كم مرة يتكرر ذلك على المقياس صفر-١٠؟ صفر= أبداً ١٠ = تقريبا بشكل مستمر
٦- شد عضلي	لا<br>نعم ←	٠ ١ ٣ ٢ ٤ ٦ ٥ ٧ ٨ ٩ ١٠	٠ ١ ٣ ٢ ٤ ٦ ٥ ٧ ٨ ٩ ١٠	٠ ١ ٣ ٢ ٤ ٦ ٥ ٧ ٨ ٩ ١٠
٧- تورم في الساقيين	لا<br>نعم ←	٠ ١ ٣ ٢ ٤ ٦ ٥ ٧ ٨ ٩ ١٠	٠ ١ ٣ ٢ ٤ ٦ ٥ ٧ ٨ ٩ ١٠	٠ ١ ٣ ٢ ٤ ٦ ٥ ٧ ٨ ٩ ١٠
٨- قصر النفس	لا<br>نعم ←	٠ ١ ٣ ٢ ٤ ٦ ٥ ٧ ٨ ٩ ١٠	٠ ١ ٣ ٢ ٤ ٦ ٥ ٧ ٨ ٩ ١٠	٠ ١ ٣ ٢ ٤ ٦ ٥ ٧ ٨ ٩ ١٠
٩- الدوار أو الدوخة	لا<br>نعم ←	٠ ١ ٣ ٢ ٤ ٦ ٥ ٧ ٨ ٩ ١٠	٠ ١ ٣ ٢ ٤ ٦ ٥ ٧ ٨ ٩ ١٠	٠ ١ ٣ ٢ ٤ ٦ ٥ ٧ ٨ ٩ ١٠
١٠- عدم ثبات الساقين ، صعوبة المحافظة على سكون وثبات الساقين	لا<br>نعم ←	٠ ١ ٣ ٢ ٤ ٦ ٥ ٧ ٨ ٩ ١٠	٠ ١ ٣ ٢ ٤ ٦ ٥ ٧ ٨ ٩ ١٠	٠ ١ ٣ ٢ ٤ ٦ ٥ ٧ ٨ ٩ ١٠
١١- تنميل أو وخز خفيف في القدمين	لا<br>نعم ←	٠ ١ ٣ ٢ ٤ ٦ ٥ ٧ ٨ ٩ ١٠	٠ ١ ٣ ٢ ٤ ٦ ٥ ٧ ٨ ٩ ١٠	٠ ١ ٣ ٢ ٤ ٦ ٥ ٧ ٨ ٩ ١٠
١٢- الشعور بالتعب أو فقدان الطاقة	لا<br>نعم ←	٠ ١ ٣ ٢ ٤ ٦ ٥ ٧ ٨ ٩ ١٠	٠ ١ ٣ ٢ ٤ ٦ ٥ ٧ ٨ ٩ ١٠	٠ ١ ٣ ٢ ٤ ٦ ٥ ٧ ٨ ٩ ١٠
١٣- الكحة (السعال)	لا<br>نعم ←	٠ ١ ٣ ٢ ٤ ٦ ٥ ٧ ٨ ٩ ١٠	٠ ١ ٣ ٢ ٤ ٦ ٥ ٧ ٨ ٩ ١٠	٠ ١ ٣ ٢ ٤ ٦ ٥ ٧ ٨ ٩ ١٠

**خلال الأسابيع الأربعة الماضية:**<br>**هل عانيت من هذا العرض؟**		**إذا نعم:**		
		**الى أي درجة أزعجتك أو ضايقتك على المقياس صفر ـ ١٠**<br>**صفر = لا يوجد**<br>**١٠ = كثيراً جداً**	**كيف كانت شدتها في العادة على المقياس صفرـ١٠؟**<br>**صفر= لا يوجد**<br>**١٠ = شديدة جداً**	**كم مرة يتكرر ذلك على المقياس صفرـ١٠؟**<br>**صفر= أبداً**<br>**١٠ = تقريبا بشكل مستمر**
١٤ـ جفاف الفم	**لا**<br>نعم ←	٠ ١ ٣ ٢ ٤ ٦ ٥ ٧ ٨ ٩ ١٠	٠ ١ ٣ ٢ ٤ ٦ ٥ ٧ ٨ ٩ ١٠	٠ ١ ٣ ٢ ٤ ٦ ٥ ٧ ٨ ٩ ١٠
١٥ـ ألم العظام أو المفاصل	**لا**<br>نعم ←	٠ ١ ٣ ٢ ٤ ٦ ٥ ٧ ٨ ٩ ١٠	٠ ١ ٣ ٢ ٤ ٦ ٥ ٧ ٨ ٩ ١٠	٠ ١ ٣ ٢ ٤ ٦ ٥ ٧ ٨ ٩ ١٠
١٦ـ ألم في الصدر	**لا**<br>نعم ←	٠ ١ ٣ ٢ ٤ ٦ ٥ ٧ ٨ ٩ ١٠	٠ ١ ٣ ٢ ٤ ٦ ٥ ٧ ٨ ٩ ١٠	٠ ١ ٣ ٢ ٤ ٦ ٥ ٧ ٨ ٩ ١٠
١٧ـ الصداع	**لا**<br>نعم ←	٠ ١ ٣ ٢ ٤ ٦ ٥ ٧ ٨ ٩ ١٠	٠ ١ ٣ ٢ ٤ ٦ ٥ ٧ ٨ ٩ ١٠	٠ ١ ٣ ٢ ٤ ٦ ٥ ٧ ٨ ٩ ١٠
١٨ـ وجع العضلات	**لا**<br>نعم ←	٠ ١ ٣ ٢ ٤ ٦ ٥ ٧ ٨ ٩ ١٠	٠ ١ ٣ ٢ ٤ ٦ ٥ ٧ ٨ ٩ ١٠	٠ ١ ٣ ٢ ٤ ٦ ٥ ٧ ٨ ٩ ١٠
١٩ـ الصعوبة في التركيز	**لا**<br>نعم ←	٠ ١ ٣ ٢ ٤ ٦ ٥ ٧ ٨ ٩ ١٠	٠ ١ ٣ ٢ ٤ ٦ ٥ ٧ ٨ ٩ ١٠	٠ ١ ٣ ٢ ٤ ٦ ٥ ٧ ٨ ٩ ١٠
٢٠ـ جفاف الجلد	**لا**<br>نعم ←	٠ ١ ٣ ٢ ٤ ٦ ٥ ٧ ٨ ٩ ١٠	٠ ١ ٣ ٢ ٤ ٦ ٥ ٧ ٨ ٩ ١٠	٠ ١ ٣ ٢ ٤ ٦ ٥ ٧ ٨ ٩ ١٠
٢١ـ الحكة	**لا**<br>نعم ←	٠ ١ ٣ ٢ ٤ ٦ ٥ ٧ ٨ ٩ ١٠	٠ ١ ٣ ٢ ٤ ٦ ٥ ٧ ٨ ٩ ١٠	٠ ١ ٣ ٢ ٤ ٦ ٥ ٧ ٨ ٩ ١٠

**خلال الأسابيع الأربعة الماضية:** **هل عانيت من هذا العرض؟**		**إذا نعم:**		
		**الى أي درجة أزعجتك أو ضايقتك على المقياس صفر ـ ١٠**<br>**صفر = لا يوجد**<br>**١٠ = كثيراً جداً**	**كيف كانت شدتها في العادة على المقياس صفر-١٠؟**<br>**صفر= لا يوجد**<br>**١٠ = شديدة جداً**	**كم مرة يتكرر ذلك على المقياس صفر-١٠؟**<br>**صفر= أبداً**<br>**١٠ = تقريبا بشكل مستمر**
٢٢ـ القلق	**لا**<br>نعم ←	٠ ١ ٣ ٢ ٤ ٦ ٥ ٧ ٨ ٩ ١٠	٠ ١ ٣ ٢ ٤ ٦ ٥ ٧ ٨ ٩ ١٠	٠ ١ ٣ ٢ ٤ ٦ ٥ ٧ ٨ ٩ ١٠
٢٣ـ الشعور بالتوتر	**لا**<br>نعم ←	٠ ١ ٣ ٢ ٤ ٦ ٥ ٧ ٨ ٩ ١٠	٠ ١ ٣ ٢ ٤ ٦ ٥ ٧ ٨ ٩ ١٠	٠ ١ ٣ ٢ ٤ ٦ ٥ ٧ ٨ ٩ ١٠
٢٤ـ صعوبة في النوم	**لا**<br>نعم ←	٠ ١ ٣ ٢ ٤ ٦ ٥ ٧ ٨ ٩ ١٠	٠ ١ ٣ ٢ ٤ ٦ ٥ ٧ ٨ ٩ ١٠	٠ ١ ٣ ٢ ٤ ٦ ٥ ٧ ٨ ٩ ١٠
٢٥ـ صعوبة في البقاء نائما"	**لا**<br>نعم ←	٠ ١ ٣ ٢ ٤ ٦ ٥ ٧ ٨ ٩ ١٠	٠ ١ ٣ ٢ ٤ ٦ ٥ ٧ ٨ ٩ ١٠	٠ ١ ٣ ٢ ٤ ٦ ٥ ٧ ٨ ٩ ١٠
٢٦ـ الشعور بسرعة الانفعال و الغضب	**لا**<br>نعم ←	٠ ١ ٣ ٢ ٤ ٦ ٥ ٧ ٨ ٩ ١٠	٠ ١ ٣ ٢ ٤ ٦ ٥ ٧ ٨ ٩ ١٠	٠ ١ ٣ ٢ ٤ ٦ ٥ ٧ ٨ ٩ ١٠
٢٧ـ الشعور بالحزن	**لا**<br>نعم ←	٠ ١ ٣ ٢ ٤ ٦ ٥ ٧ ٨ ٩ ١٠	٠ ١ ٣ ٢ ٤ ٦ ٥ ٧ ٨ ٩ ١٠	٠ ١ ٣ ٢ ٤ ٦ ٥ ٧ ٨ ٩ ١٠
٢٨ـ الشعور بالاضطراب وعدم راحة البال	**لا**<br>نعم ←	٠ ١ ٣ ٢ ٤ ٦ ٥ ٧ ٨ ٩ ١٠	٠ ١ ٣ ٢ ٤ ٦ ٥ ٧ ٨ ٩ ١٠	٠ ١ ٣ ٢ ٤ ٦ ٥ ٧ ٨ ٩ ١٠
٢٩ـ الاكتئاب	**لا**<br>نعم ←	٠ ١ ٣ ٢ ٤ ٦ ٥ ٧ ٨ ٩ ١٠	٠ ١ ٣ ٢ ٤ ٦ ٥ ٧ ٨ ٩ ١٠	٠ ١ ٣ ٢ ٤ ٦ ٥ ٧ ٨ ٩ ١٠

**<u>خلال الأسابيع الأربعة الماضية:</u>**<br>**هل عانيت من هذا العرض؟**		**<u>إذا نعم:</u>**		
		**الى أي <u>درجة أزعجتك</u> أو <u>ضايقتك</u> على المقياس صفر ـ ١٠**<br>**صفر = لا يوجد**<br>**١٠ = كثيراً جداً**	**كيف كانت <u>شدتها في العادة</u> على المقياس صفر-١٠؟**<br>**صفر= لا يوجد**<br>**١٠ = شديدة جداً**	**كم مرة <u>يتكرر</u> ذلك على المقياس صفر-١٠؟**<br>**صفر= أبداً**<br>**١٠ = تقريبا بشكل مستمر**
٣٠ـ نقص الرغبة الجنسية	**لا**<br>نعم ←	١٠ ٩ ٨ ٧ ٥ ٦ ٤ ٢ ٣ ١ ٠	١٠ ٩ ٨ ٧ ٥ ٦ ٤ ٢ ٣ ١ ٠	١٠ ٩ ٨ ٧ ٥ ٦ ٤ ٢ ٣ ١ ٠
٣١ـ صعوبة في الإثارة الجنسية	**لا**<br>نعم ←	١٠ ٩ ٨ ٧ ٥ ٦ ٤ ٢ ٣ ١ ٠	١٠ ٩ ٨ ٧ ٥ ٦ ٤ ٢ ٣ ١ ٠	١٠ ٩ ٨ ٧ ٥ ٦ ٤ ٢ ٣ ١ ٠
٣ ٢ ـ كثره تكرار التبول في الليل (أثناء النوم)	**لا**<br>نعم ←	١٠ ٩ ٨ ٧ ٥ ٦ ٤ ٢ ٣ ١ ٠	١٠ ٩ ٨ ٧ ٥ ٦ ٤ ٢ ٣ ١ ٠	١٠ ٩ ٨ ٧ ٥ ٦ ٤ ٢ ٣ ١ ٠
**اذا كان لديك أي أعراض أخرى خلال الأسابيع الأربعة الماضية, من فضلك اذكرها أدناه:**				
اخرى:	**لا**<br>نعم ←	١٠ ٩ ٨ ٧ ٥ ٦ ٤ ٢ ٣ ١ ٠	١٠ ٩ ٨ ٧ ٥ ٦ ٤ ٢ ٣ ١ ٠	١٠ ٩ ٨ ٧ ٥ ٦ ٤ ٢ ٣ ١ ٠
اخرى:	**لا**<br>نعم ←	١٠ ٩ ٨ ٧ ٥ ٦ ٤ ٢ ٣ ١ ٠	١٠ ٩ ٨ ٧ ٥ ٦ ٤ ٢ ٣ ١ ٠	١٠ ٩ ٨ ٧ ٥ ٦ ٤ ٢ ٣ ١ ٠
اخرى:	**لا**<br>نعم ←	١٠ ٩ ٨ ٧ ٥ ٦ ٤ ٢ ٣ ١ ٠	١٠ ٩ ٨ ٧ ٥ ٦ ٤ ٢ ٣ ١ ٠	١٠ ٩ ٨ ٧ ٥ ٦ ٤ ٢ ٣ ١ ٠

شكراً لمشاركتك في اكمال هذا الإستبيان
